# Aminophosphate precursors for the synthesis of near‐unity emitting InP quantum dots and their application in liver cancer diagnosis

**DOI:** 10.1002/EXP.20220082

**Published:** 2022-07-15

**Authors:** Yanbin Zhang, Yanbing Lv, Lin‐Song Li, Xue‐Jie Zhao, Mei‐Xia Zhao, Huaibin Shen

**Affiliations:** ^1^ Key Laboratory of Natural Medicine and Immuno‐Engineering of Henan Province Henan University Kaifeng China; ^2^ Key Laboratory for Special Functional Materials of Ministry of Education School of Materials and Engineering Henan University Kaifeng China

**Keywords:** bioimaging, InP quantum dots, in vitro diagnostics, liver cancer, shell engineering

## Abstract

InP quantum dots (QDs) are a promising and environment‐friendly alternative to Cd‐based QDs for in vitro diagnostics and bioimaging applications. However, their poor fluorescence and stability severely limit their biological applications. Herein, we synthesize bright (∼100%) and stable InP‐based core/shell QDs by using cost‐effective and low‐toxic phosphorus source, and then aqueous InP QDs are prepared with quantum yield over 80% by shell engineering. The immunoassay of alpha‐fetoprotein can be detected in the widest analytical range of 1–1000 ng ml^−1^ and the limit of detection of 0.58 ng ml^−1^ by using those InP QDs‐based fluorescent probes, making it the best‐performing heavy metal‐free detection reported so far, comparable to state‐of‐the‐art Cd‐QDs‐based probes. Furthermore, the high‐quality aqueous InP QDs exhibit excellent performance in specific labeling of liver cancer cells and in vivo tumor‐targeted imaging of live mice. Overall, the present work demonstrates the great potential of novel high‐quality Cd‐free InP QDs in cancer diagnosis and image‐guided surgery.

## Graphical abstract

Environment‐friendly InP core/shell quantum dots (QDs) with quantum yield of ∼100% are prepared by aminophosphate precursors. The limit of detection of alpha‐fetoprotein for in vitro diagnostics, specific labeling of liver cancer cells, and in vivo tumor‐targeted imaging of live mice are comparable to that of the state‐of‐the‐art Cd‐QDs‐based probes.

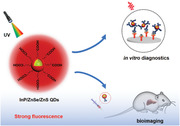

## 1 INTRODUCTION

Quantum dots (QDs) are considered as a promising fluorescent probe for diagnostics and bioimaging applications owing to their unique optical features, including tunable emission wavelength, high photoluminescence quantum yield (PL QY), and excellent stability.^[^ ^1^, ^2^, ^3^, ^4^, ^5^ ^]^ Since the heavy metal Cd ions in the state‐of‐the‐art CdSe QDs are potentially harmful to the living organisms even if they are coated with nontoxic ZnS shell,^[^ ^6^, ^7^, ^8^ ^]^ the development of alternative luminescent materials has received considerable research attention.^[^ ^9^, ^10^ ^]^ The optical properties of InP QDs are similar to those of the traditional CdSe QDs, which renders them an effective substitute material.^[^ ^9^, ^11^, ^12^, ^13^ ^]^ Although there have been reports on the synthesis of InP‐based QDs with a PL QY of over 90%, their application in biological field has been limited due to the complex, low‐stability, and hazardous synthesis and surface ligand exchange process.^[^ ^13^, ^14^, ^15^ ^]^ Therefore, a safe and facile route to prepare InP‐based QDs with superior optical properties is in urgent demand.

Another key issue in the use of InP QDs for biological applications is the drastic loss of PL QY during the phase transfer process. This can be mainly attributed to the surface oxidation which can easily occur as compared to the Cd‐containing counterparts.^[^ ^16^, ^17^, ^18^ ^]^ An effective strategy for improving the stability of InP QDs is to coat a thick large bandgap ZnS shell, which not only isolates the InP core from the environment, but also strongly confines the carriers inside the QDs.^[^ ^19^, ^20^ ^]^ Unfortunately, the growth of a thick ZnS shell on the InP cores remains challenging due to the large lattice mismatch (7.7%) between InP (5.87 Å) and ZnS (5.41 Å).^[^ ^12^, ^21^ ^]^ During the phase transfer process using ligand exchange method, the PL QY decreases sharply because 3‐mercaptopropionic acid (3‐MPA) can etch the surface of QDs and seriously aggravate the carrier leakage process.^[^ ^22^, ^23^ ^]^ Further, the absence of suitable hydrosoluble phosphorous precursors makes it impossible to directly synthesize InP QDs in aqueous solution similar to the microwave synthesis of water‐dispersed CdTe core/shell QDs.^[^ ^24^ ^]^ To resolve this issue, a suitable phase transfer scheme is required to synthesize aqueous InP QDs, which is of great significance in biological applications.

To address these challenges, we synthesize high‐quality InP/ZnSe/ZnS core/shell/shell QDs with near‐unity PL QY by using tris(dimethylamino)phosphines ((DMA)_3_P) as the phosphorus source and propose a two‐step strategy to obtain bright and stable InP‐QDs bio‐probes with 3‐MPA as the functional ligand. Specifically, the initial thermodynamic growth is used to obtain thin‐shell QDs with high fluorescence; subsequently, the additional ZnS shell is kinetically grown in an aqueous solution. The kinetic growth avoids the generation of lattice defects induced by the release of lattice strain in the traditional thick shell growth process as well as facilitates the aqueous phase transfer. The InP/ZnSe/ZnS//ZnS QDs probes are prepared by coupling the alpha‐fetoprotein (AFP, serum markers of hepatocellular carcinoma [HCC]) antibody to quantify AFP antigen by using the QDs‐based fluorescence‐linked immunosorbent assay (QDs‐FLISA). The limit of detection (LOD) reaches as low as 0.58 ng ml^−1^. In addition, these QDs probes are used to target HepG2 cells (a HCC cell line that can positively express the AFP). They are also applied for in vivo active tumor targeting and sensitive imaging of liver cancer. The synergistic use of InP QDs for in vivo bioimaging and in vitro diagnostics can increase the accuracy of liver cancer diagnosis. These findings demonstrate the immense potential of the proposed high‐quality nontoxic QDs bio‐probes for diagnostics and other biomedical applications.

## 2 RESULTS AND DISCUSSION

### 2.1 Synthesis of red near‐unity emitting InP/ZnSe/ZnS QDs based on aminophosphate precursors

**FIGURE 1:**
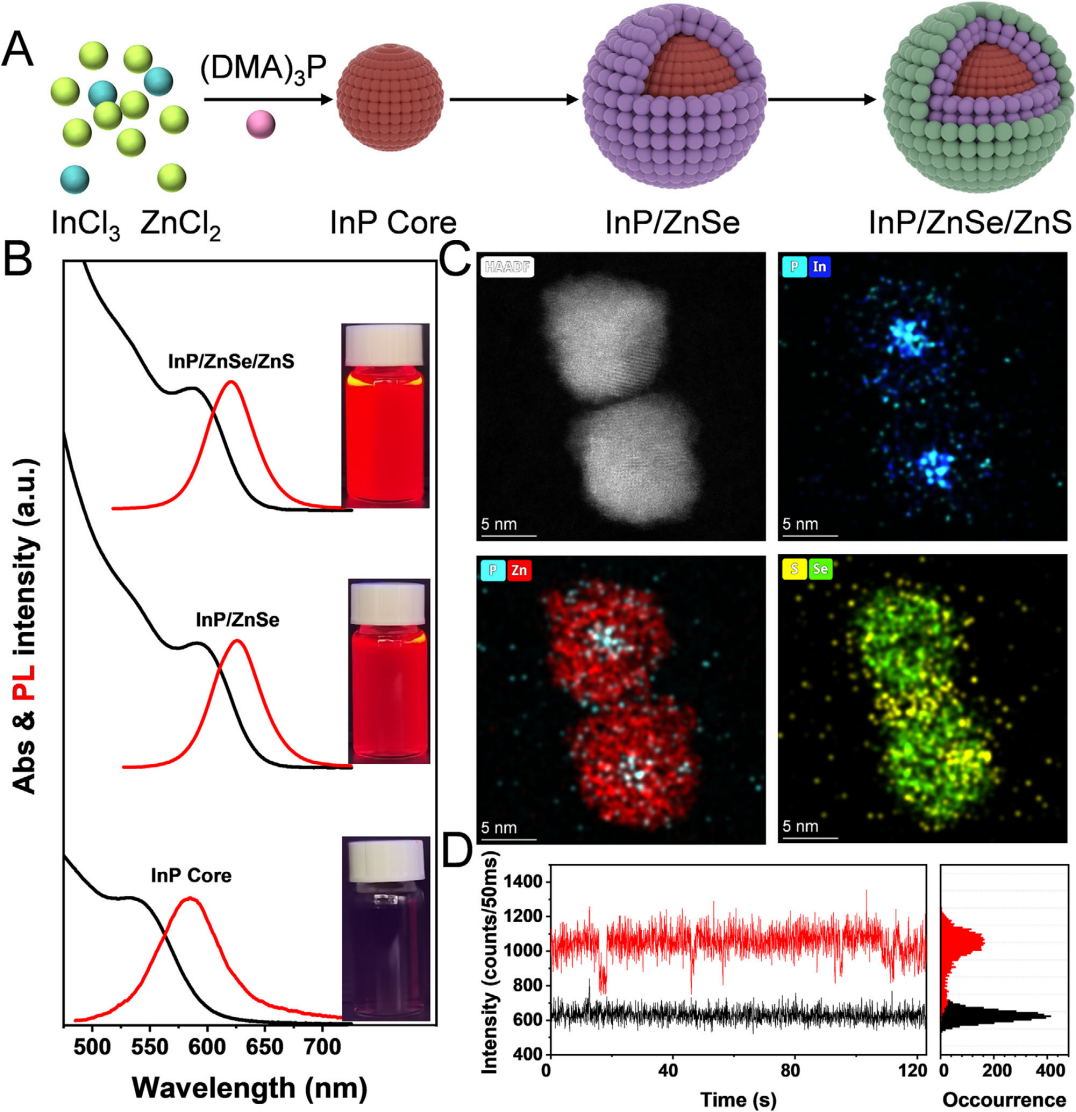
(A) Synthesis scheme of highly luminescent InP/ZnSe/ZnS QDs with aminophosphate precursors; (B) Evolution of absorption spectra (black) and PL spectra (red) during the synthesis of QDs. Insets show the photographs of QDs at different synthesis stages under UV light; (C) HR‐TEM image and EDS elemental maps of In, P, Zn, Se, and S elements in InP/ZnSe/ZnS core/shell QDs; (D) PL intensity trajectory of single InP/ZnSe/ZnS core/shell QDs. The red and black traces are the PL intensities of a single QD and the corrected background noise intensities, respectively

For the first time, we have synthesized InP/ZnSe/ZnS core/shell QDs with near‐unity PL QY (∼100%) using a method, which is different from the previously reported methods in two main aspects. First, InP core QDs are prepared by using a cost‐effective and low‐toxicity phosphorus source ((DMA)_3_P) as an alternative precursor to highly toxic and expensive tris(trimethylsilyl) phosphine.^[^ ^25^, ^26^, ^27^ ^]^ Second, ZnCl_2_ is used as the precursor instead of zinc carboxylate in the entire synthesis process (Figure 1A). The traditional method of using carboxylate as the precursor is abandoned, which is conducive to eliminate the influence of defects caused by oxidation of InP core surface (Figure S1A), and the use of HF which with serious corrosivity and toxicity is avoided in the post‐processing stage.^[^ ^13^, ^28^, ^29^, ^30^ ^]^ This acid‐free method provides a low oxygen and water environment for subsequent growth of InP shell (Figure S1B), which is beneficial to improve the PL QY and reduce the full width of half‐maximums (FWHM) of InP core/shell QDs (Figure S2A,B). Figure 1B presents the evolution of the ultraviolet‐visible (UV–vis) absorption and PL emission spectra of InP/ZnSe/ZnS QDs upon shell growth. When growing the ZnSe, both the first exciton absorption peak and the PL peak are shifted toward lower energy, and this red‐shift is attributed to the weaker electron confinement ability of ZnSe for InP.^[^ ^12^ ^]^ The wide bandgap ZnS layer is further grown to confine the excitons, and red InP core/shell QDs with near‐unity emission are obtained. The high‐resolution transmission electron microscopy (HR‐TEM) image clearly reveals continuous lattice fringes throughout the QDs, indicating no lattice defects (Figure 1C). The energy dispersive spectroscopy (EDS) mapping of the five elements (i.e., P, In, Zn, S, and Se) is shown in Figure 1C, which confirms the epitaxial growth of ZnSe/ZnS layers on the InP core. Meanwhile, the ZnSe/ZnS double shell layer effectively eliminates the trapping of excited charge carriers and suppresses the emission blinking of QDs (Figure S3 and Figure 1D).^[^ ^15^ ^]^

### 2.2 Creation of a thicker ZnS shell by kinetic growth while achieving phase transfer

**FIGURE 2:**
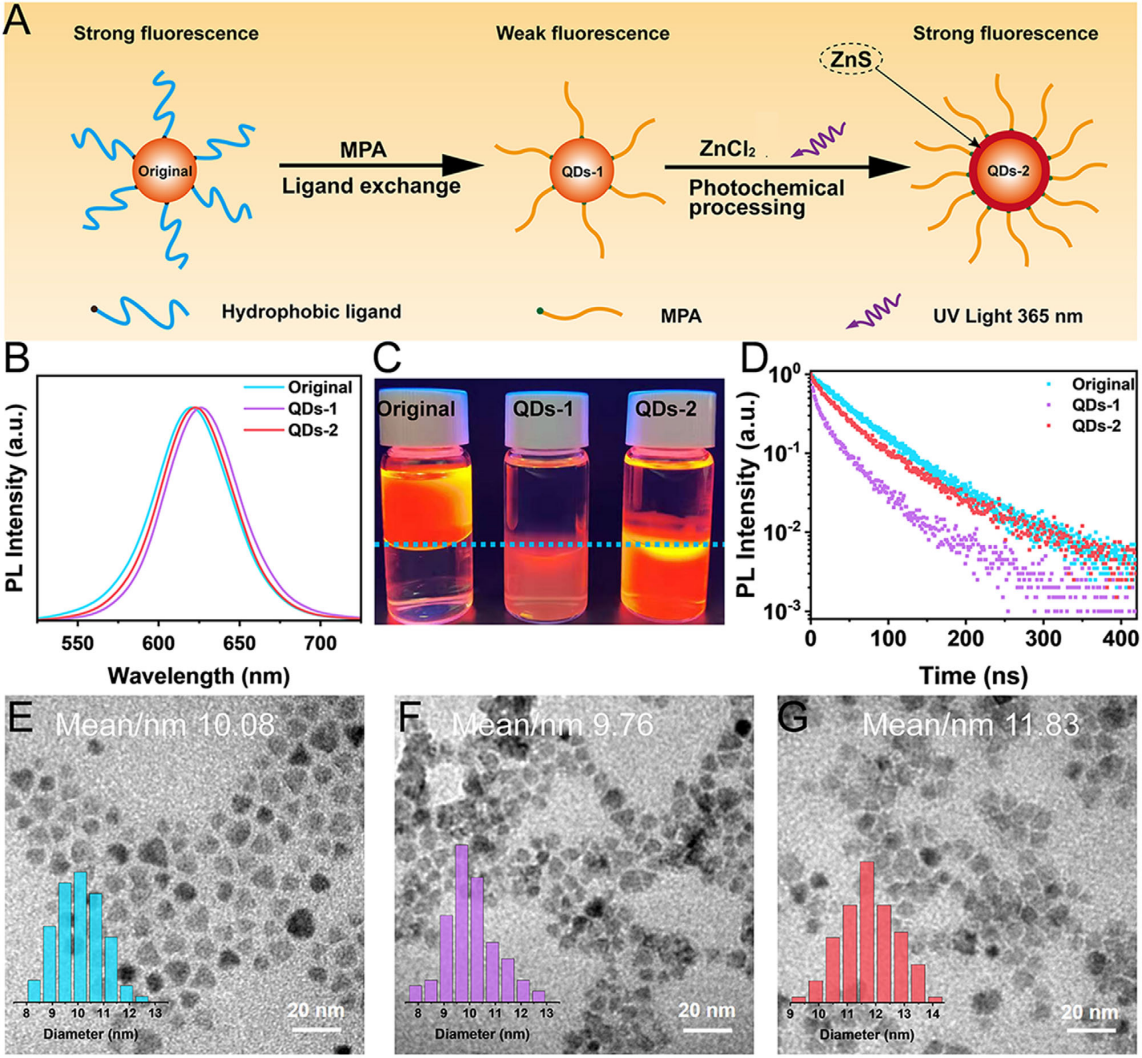
(A) Schematic representation of the synthesis of highly luminescent InP QDs in an aqueous solution. It consists of two steps: (1) ligand exchange and (2) photochemical processing; (B) fluorescence emission spectra; (C) photographs under 365 nm UV light; (D) PL lifetimes of QDs; (E–G) TEM images of the three samples, and the insets show the corresponding size distributions

The bright and stable luminescence of InP QDs in aqueous solution is necessary to realize highly sensitive in vitro diagnostics and in vivo bioimaging. To meet this requirement, a photochemical processing step is added to the simple ligand exchange process, which significantly improves the PL intensity and stability of InP QDs in aqueous solution (as shown in Figure 2A). It has been reported that 3‐MPA can decompose S^2–^ under UV irradiation or prolonged heating.^[^ ^23^, ^31^ ^]^ We take advantage of this property by adding ZnCl_2_ in the aqueous solution so that the decomposed S^2–^ can combine with Zn^2+^, resulting in the growth of a thicker ZnS layer in the aqueous solution. To accelerate the ligand exchange process, the reaction temperature in the phase transfer process is set at 60–70°C. This heat‐assisted strategy greatly shortens the reaction time for the preparation of aqueous InP QDs. During this process, thermal assistance and UV irradiation induce the decomposition of S^2–^ from 3‐MPA, which binds to Zn^2+^ in the solution and leads to the kinetic growth of ZnS shells. This combined thermodynamic and kinetic growth approach for the preparation of aqueous InP QDs have two significant advantages: (1) this method compensates for the loss of fluorescence due to ligand exchange. In other words, it can maintain the optical properties of InP QDs in aqueous solution comparable to those in organic solvents; (2) this method involves a large number of surface ligands, which not only help to maintain good dispersion of InP QDs in the aqueous solution, but also maximize the coupling efficiency of InP QDs with antibodies. For simplicity, we denote the InP QDs obtained in the organic solvents as “original,” the aqueous InP QDs obtained by simple ligand exchange as “QDs‐1,” and the InP QDs obtained by photochemical processing as “QDs‐2.”

Figure 2B demonstrates the fluorescence spectra of InP QDs after ligand exchange and photochemical processing. It may be noted that the PL peak position of both QDs‐1 and QDs‐2 is slightly red‐shifted as compared to that of the original QDs. The PL peak position of QDs‐1 is more significantly red‐shifted, which may be attributed to the poor dispersion in aqueous solution caused by the ligand exchange and the resonance energy transfer caused by the agglomeration of particles. This can also be confirmed by the TEM. It can be seen from Figure 2E–G that QDs‐1 exhibits some agglomeration in aqueous solution after ligand exchange, while QDs‐2 showed a relatively uniform dispersion after UV irradiation. Furthermore, the average size of QDs‐1 decreases by 0.3 nm, and the corresponding elemental map also indicates that the Zn/In ratio of QDs‐1 decreases (Figure S4A,B), which is presumably due to the surface etching of QDs by 3‐MPA.^[^ ^22^ ^]^ By contrast, the average size of QDs‐2 increases by ∼2 nm after UV irradiation, indicating the successful growth of a thicker ZnS layer (∼1 nm) in the aqueous solution. The corresponding elemental map also shows an increase in the Zn/In ratio of QDs‐2 (Figure S4C). Further, the rapid increase in the S/P ratio indicates an increase in the sulfhydryl groups on the surface of QDs. As shown in Figure S5, the zinc‐blende crystal structure of InP QDs is maintained during the shell growth of ZnS. The diffraction peaks shift toward higher angles, indicating that the ZnS shell is successfully grown in an epitaxial way. Furthermore, inductively coupled plasma (ICP) elemental analysis confirms this interpretation, and the proportion of Zn elements in QDs‐2 increases significantly after UV irradiation (Table S1). All these findings confirm the successful kinetic growth of a thicker ZnS layer outside the QDs in the aqueous solution.

Fluorescence decay is one of the most common problems of QDs during the ligand exchange process. This is mainly because the shedding of ligand molecules inevitably leads to defects on the surface of QDs,^[^ ^32^ ^]^ and 3‐MPA also etches the surface of QDs, which further aggravates these defects.^[^ ^22^ ^]^ Consequently, the excitons of QDs are captured by the surface defects and the occurrence probability of non‐radiative recombination increases, leading to a severe decrease in the fluorescence intensity.^[^ ^33^, ^34^ ^]^ It can be seen in Figure 2C that the PL QY of QDs‐1 is severely deteriorated (PL QY reduces to 25%), which is consistent with the previous reports.^[^ ^32^ ^]^ After growing ZnS, the PL QY of QDs‐2 is significantly enhanced relative to that of QDs‐1 (PL QY recovers to 81%). The experimental setup is shown in Figure S6A. It is clear from Figure S6B that QDs‐2 has the highest fluorescence at 40 min of UV irradiation, and continued irradiation leads to a decrease in the fluorescence. To this end, we speculate that there are two competing mechanisms on the surface of QDs: (1) the growth of ZnS layer can significantly increase the PL intensity, and (2) high‐power UV irradiation accelerates the photoetching on the surface of QDs, causing a decrease in the PL intensity. Therefore, at 40 min of UV irradiation, the re‐grown ZnS shell is sufficient to eliminate the surface defects, where the brightest fluorescence is obtained, and continued irradiation causes a decrease in the fluorescence. For comparison, we have tested the changes in the PL intensity without UV irradiation and by replacing ZnCl_2_ with CaCl_2_ (as shown in Figure S6C–E). It is observed that the PL intensity remains basically unchanged under the condition of no UV irradiation. However, after replacing CaCl_2_ with ZnCl_2_ and under UV irradiation, the PL intensity of QDs decreases significantly. This confirms the proposed competing mechanisms, suggesting the requirement for rapid growth of the outermost ZnS shell to ensure the maximization of PL intensity.

Next, we have investigated the effect of surface modification on the carrier dynamics using time‐resolved photoluminescence (TRPL) spectroscopy (Figure 2D). It is clear that the fluorescence lifetime of the QDs‐1 samples after ligand exchange exhibits a significant decay compared to that of the QDs in organic solvents, which may be due to the trapping of surface defect states. By contrast, the fluorescence lifetime of QDs‐2 samples recovers significantly, indicating that the thicker ZnS layer grown by the kinetic growth strategy sufficiently passivates the surface traps caused by ligand exchange.^[^ ^14^, ^15^, ^35^, ^36^ ^]^ This is crucial to maintain the optical properties of the QDs in aqueous solution. 3‐MAP has both sulfhydryl and carboxyl groups, so a large amount of 3‐MPA on the surface can passivate the surface traps to a large extent, thereby greatly reducing the possibilities of exciton capture by surface defects and non‐radiative recombination. Therefore, a large amount of 3‐MPA on the surface can enhance the fluorescence of QDs, which is consistent with the observed weak fluorescence of QDs‐1 versus the strong fluorescence of QDs‐2.

### 2.3 Surface ligands of InP QDs

The Fourier transform infrared (FTIR) spectra in Figure S7A indicate that the original organic ligands on the surface of both QDs‐1 and QDs‐2 are successfully replaced by 3‐MPA. Dynamic light scattering shows that the hydrodynamic diameter of QDs‐2 is smaller because more 3‐MPA is adsorbed on their surface (Figure S7B).^[^ ^37^ ^]^ The protein dispersibility indices (PDI) of QDs‐1 and QDs‐2 are 0.223 and 0.169, respectively, indicating that the dispersibility of QDs‐2 in aqueous solution is better than that of QDs‐1.^[^ ^37^, ^38^ ^]^ At the same QD concentration and pH value, the zeta potentials of QDs‐1 and QDs‐2 are −24.9 and −50.7 mV, respectively (Figure S7C). The absolute value of zeta potential is greater than 30 mV, suggesting a strong static repulsion between the particles and a good colloidal stability of QDs‐2.^[^ ^37^ ^]^ Therefore, QDs‐2 exhibits better dispersion and stability in aqueous solution, so it is more suitable for biological applications.

Another reason for QDs‐2 to maintain such excellent optical properties in aqueous solution is the higher number (i.e., density) of surface ligands. This is important for the subsequent coupling of more bio‐functional molecules in biological applications.^[^ ^39^, ^40^ ^]^ By analyzing the ligand density on the surface of QDs using thermal gravimetric analysis (TGA) tests, as shown in Figure S8, it is observed that the mass fractions decrease to a certain extent when the temperature increases. The surface ligand densities of QDs‐1 and QDs‐2 are calculated to be 1.57 and 5.36 ligands per nm^−2^, respectively (see Supporting Information for details).^[^ ^41^ ^]^ This is consistent with the previously reported order of magnitude.^[^ ^22^, ^37^ ^]^ The higher density of ligands on the surface of the QDs‐2 sample also explains the smaller hydrodynamic size with the larger absolute value of the zeta potential, which is consistent with the above data.

### 2.4 Stability of InP QDs in aqueous solution

**FIGURE 3:**
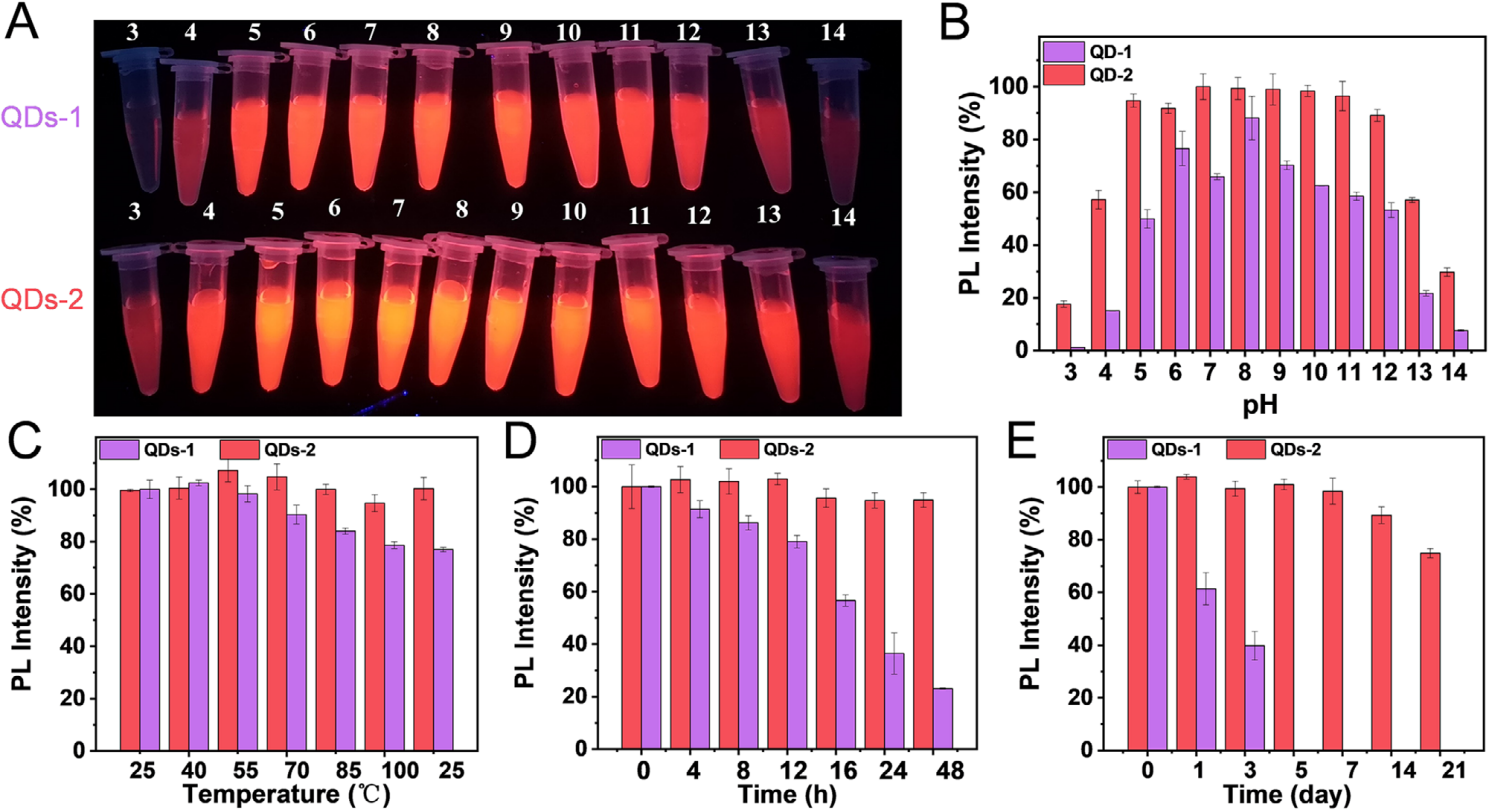
Stability of aqueous QDs: (A) and (B) stability at different pH values, (C) thermal stability, (D) photostability, and (E) stability at 4°C versus storage time

Another advantage of the large ligand density of QDs‐2 is that they can maintain their dispersion and stability in aqueous solutions. To verify this conclusion, we analyzed the stability of aqueous QDs. Figure 3A,B shows the PL intensity variation of both the samples in acidic and alkaline environments with pH values in the range of 1−14. As shown in Figure 3C, the PL intensity of QDs‐1 decreases with the increase in temperature, while that of QDs‐2 remains basically unaffected. Furthermore, the PL intensity of QDs‐1 does not return to its initial value when the temperature becomes 25°C. The results of the photostability test on the aqueous QDs are shown in Figure 3D. The PL intensity of QDs‐2 remains higher than 94% of the initial value using 365 nm UV lamp (38 W) for 48 h continuously, while that of QDs‐1 decreases to 23%.

Subsequently, the preservation time of the aqueous QDs has been evaluated, and it can be seen in Figure 3E that the PL intensity of QDs‐2 is maintained at 74% of the initial value after 21 days of preservation in a refrigerator at a temperature of 4°C. Furthermore, it is worth mentioning that QDs‐1 exhibit agglomeration and sedimentation after 3 days, and the PL intensity decreases significantly. Such a huge difference in the stability of the two samples in aqueous solution may be ascribed to the shedding of 3‐MPA on the surface. On the one hand, there are fewer ligands on the surface of QDs‐1, resulting in less negative charges on the surface (Figure S7C), which cannot maintain long‐term stability by electrostatic repulsion between the particles. On the other hand, fewer ligands cannot well passivate the surface defects, which in turn affects the photochemical bleaching resistance of QDs.

### 2.5 Sensitive detection of AFP antigen based on QDs‐FLISA

The accurate quantification of AFP antigen is of great significance for the early diagnosis of HCC.^[^ ^42^ ^]^ Currently, various QDs‐based analytical techniques are widely used in the AFP assay, providing valuable references for early screening and observation of recovery progress during the treatment period. Among them, the QDs‐FLISA (the detection principle is shown in Figure S9) is an accurate and sensitive technique for the in vitro diagnostics of AFP antigen, and its detection sensitivity can be an order of magnitude higher than that of the traditional colloidal gold method.

Here, we used QDs‐1 and QDs‐2 for the quantitative detection of AFP based on the QDs‐FLISA. The free ‐COOH on the surface of the aqueous QDs were coupled to the ‐NH_2_ containing AFP antibody under the combined activation of EDC (1‐ethyl‐3‐(3‐(dimethylamino) propyl) carbodiimide) and sulfo‐NHS (N‐hydroxysulfosuccinimide) to form the QDs‐AFP‐Ab probe. After the addition of EDC and sulfo‐NHS, we observed a significant aggregation of QDs‐1, which is because the addition of EDC activates the ‐COOH on the surface of the QDs, causing a decrease in the electrostatic repulsion between the QDs. Nevertheless, the dispersion of QDs‐2 is better than that of QDs‐1, which again confirms the stable performance of QDs‐2 (Figure S10).

**FIGURE 4:**
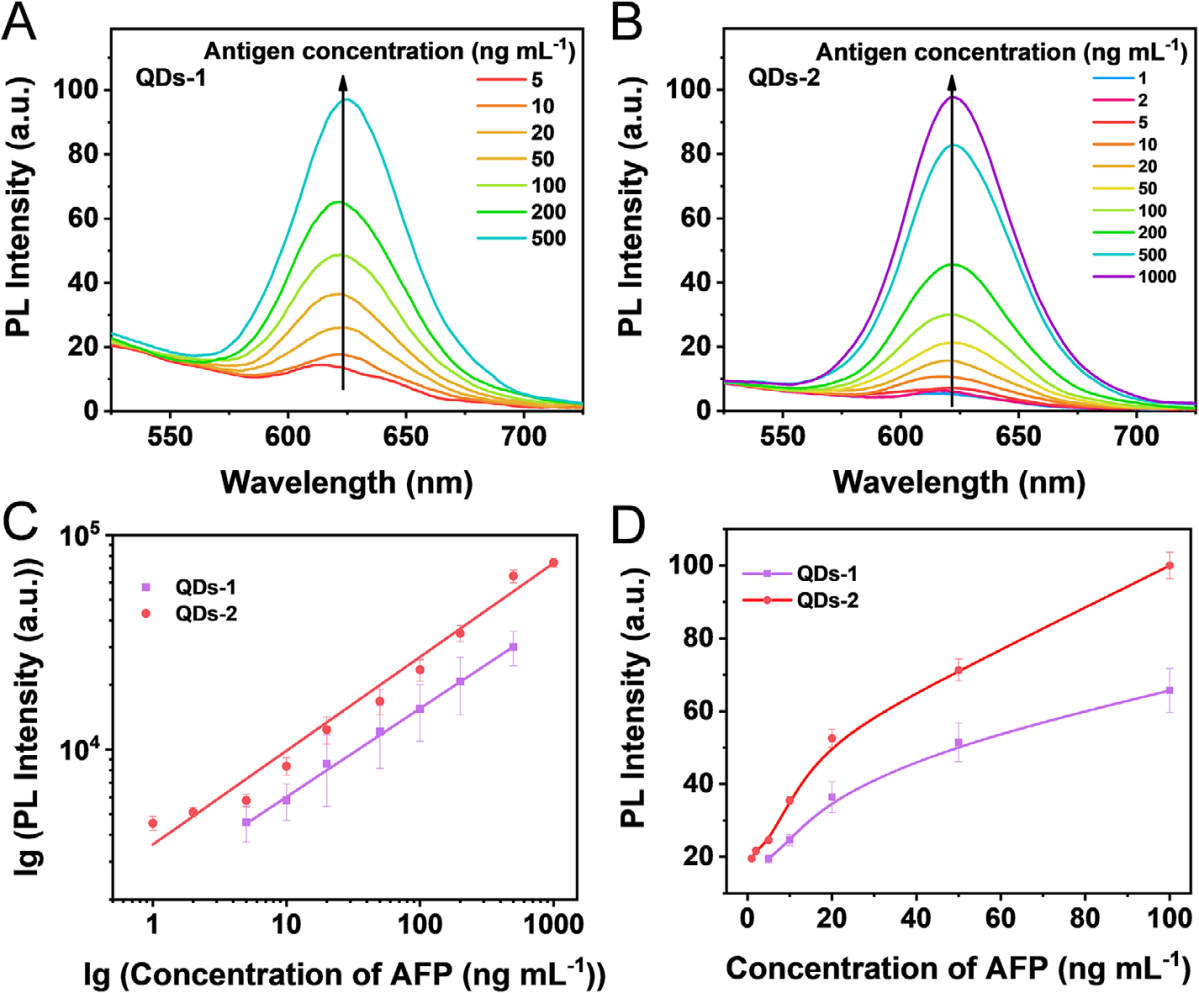
PL spectra obtained using the QDs‐FLISA method for the determination of AFP antigens by (A) QDs‐1 and (B) QDs‐2. (C) Corresponding calibration curves for quantitative detection of AFP in the concentration range of 0–1000 ng ml^−1^, and (D) calibration curves at low concentration (0–100 ng ml^−1^) of AFP antigens. The error bars indicate the standard deviations of three independent experiments

Next, we designed QDs‐AFP‐Ab probes based on aqueous QDs‐1 and QDs‐2, and the PL intensity corresponding to each AFP sample at different concentrations is shown in Figure 4. In general, the PL intensity of both the samples gradually increases with the increase in the AFP antigen concentrations (Figure 4A,B). It is noteworthy that the PL intensity of QDs‐2 is much higher than that of QDs‐1 throughout the detection range. At AFP antigen concentrations below 5 ng ml^−1^, the PL signal of QDs‐1 probe is low and cannot be distinguished from the negative wells (0 ng ml^−1^), and the detected PL intensity decreases at AFP antigen concentration of 1000 ng ml^−1^. Thus, the detection range for AFP sample concentration is 5–500 ng ml^−1^ by using QDs‐1 probe, the standard curve is lgY = 3.36 + 0.412 lgX with *R* ^2^ = 0.998 (*n* = 3) (X is AFP standard antigen concentration, Y is corresponding PL intensity, and R is linear correlation). By contrast, the PL intensity of QDs‐2 probe is linearly correlated with the AFP antigen concentration in the range of 1–1000 ng ml^−1^, with the calibration curve of lgY = 3.55 + 0.436 lgX (*R* ^2^ = 0.986, *n* = 3) in Figure 4C,D. The detection performance of these QDs‐based probes can be quantified by the LOD, which is an important parameter in the QDs‐FLISA method to characterize the detection sensitivity. The LOD of QDs‐1 antibody probe is 3.38 ng ml^−1^, while that of QDs‐2 antibody probe is 0.58 ng ml^−1^, indicating approximately 5.8‐fold improvement in the detection sensitivity (the detailed LOD determination procedure is presented in the Supporting Information). Highly sensitive detection of AFP is essential for the early diagnosis of cancer, and the level of AFP in normal human serum is less than 25 ng ml^−1^; however, when HCC occurs, the level of AFP increases dramatically. Usually, 400 ng ml^−1^ is the standard value, above which the possibility of carcinoma is considered. In addition, QDs‐2 can be used as a highly efficient fluorescent probe for quantitative immunoassays with a wider detection range and higher detection sensitivity than the other commonly used probes (Table S2).

### 2.6 Cytotoxicity of intracellular InP QDs probes

To evaluate the biocompatibility of both QDs probes (QDs‐1‐AFP‐Ab and QDs‐2‐AFP‐Ab), we examined their cytotoxicity by MTT (3‐(4,5‐dimethyl‐2‐thiazol)‐2,5‐diphenyltetrazolium bromide) assay. As shown in Figure S11, both the probes do not exhibit significant toxicity, and the cell viability remains around 60% even when incubated at high concentrations (500 μg ml^−1^). These results indicate that InP QDs have good biocompatibility and can be used as a nontoxic and safe probe for bioimaging and other biological applications.

### 2.7 In vitro and in vivo bioimaging

**FIGURE 5:**
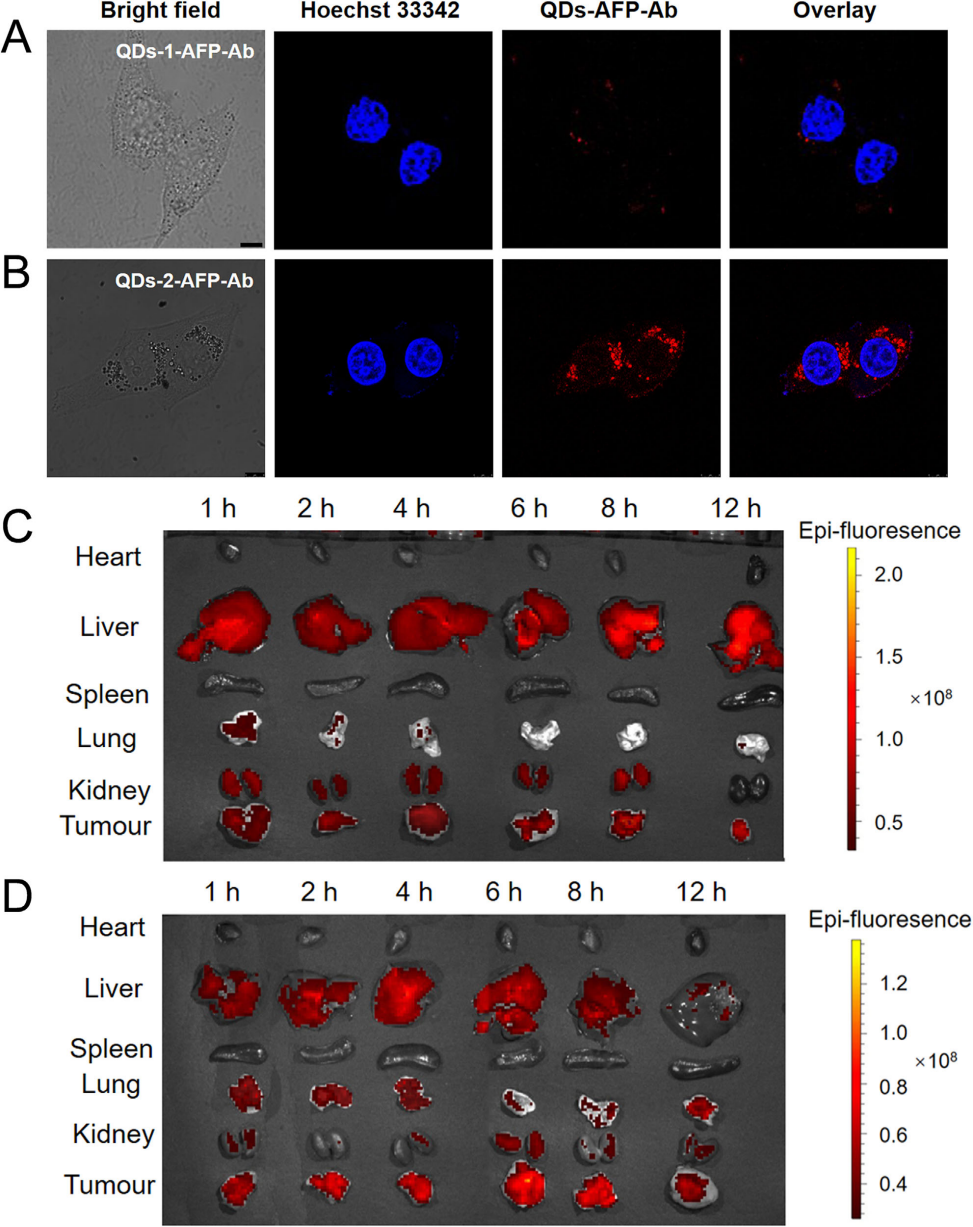
In vitro imaging of cancer cells by (A) QDs‐1‐AFP‐Ab probes and (B) QDs‐2‐AFP‐Ab probes. The nuclei are counterstained blue with Hoechst 33342. Scale bar: 8 μm. Fluorescence images of (C) QDs‐1‐AFP‐Ab probes and (D) QDs‐2‐AFP‐Ab probes in various organs of mice at different durations. The QDs probes are injected at a concentration of 5 mg kg^−1^ and the injection volume is 200 μl

To evaluate whether the QDs probe can effectively target HCC cells, the probe was co‐incubated with primary HCC HepG2 cells for 6 h and then observed by a fluorescence inverted microscope and confocal laser scanning microscope (CLSM). It can be seen in Figure S12 and Figure 5A that the QDs‐1‐AFP‐Ab probes exhibit minor aggregation around the nucleus and extremely weak fluorescence. By contrast, the strong red signal emitted in the cytoplasm of the HCC cells in Figure 5B indicates that the QDs‐2‐AFP‐Ab probes bind to the AFP antigen specifically and accurately and maintain a very strong fluorescence. This indicates that the specific uptake of QDs‐1 is much weaker than that of QDs‐2, which is mainly attributed to the following two reasons. On the one hand, QDs‐1 probe itself have fewer ligands on its surface, resulting in a limited number of coupled AFP antibodies; on the other hand, it cannot maintain long‐term stability in aqueous solution due to the fewer ligands, resulting in poor stability. The control studies using MCF‐7 cells, an AFP negative human breast cancer cell line, also show the absence of QDs binding (Figure S13). These results indicate that the QDs‐2‐AFP‐Ab probes retain their AFP binding activity and specificity.

To examine the effectiveness of QDs‐AFP‐Ab probes for in vivo targeting of tumors, the probe was injected from the tail vein into a tumor‐bearing nude mice, and the images of tumors and various organs were obtained at different durations using a small animal live imaging system (IVIS Lumina XRMS Series III). As shown in Figure 5C, QDs‐1 probe poorly targets the tumor and detects a weak fluorescent signal. By contrast, the QDs‐2 probe shows bright fluorescence and the PL intensity displays a peak after 6 h (Figure 5D). The time‐dependent enhancement of PL signal at the tumor site may be attributed to the active and specific targeting of liver cancer by the QDs probes.^[^ ^43^, ^44^, ^45^ ^]^ The corresponding 6 h images clearly show QDs‐2 at the entire tumor site, and the QDs probes mainly aggregate in the liver and tumor sites with no obvious PL signal in the heart, spleen, lung, and other organs, indicating that it has a good specific targeting effect on liver cancer. Overall, we hope that the proposed probe can serve as an accurate and effective reference for the detection and resection of HCC lesions in the future.

## 3 CONCLUSION

In this work, we established a two‐step strategy to synthesize bright and stable InP/ZnSe/ZnS QDs bio‐probes. Initially, the InP/ZnSe/ZnS QDs were obtained through thermodynamic shell growth for obtaining a high fluorescence. Subsequently, a thicker ZnS shell was kinetically grown in an epitaxial way. The results revealed that the synthesized InP/ZnSe/ZnS//ZnS QDs exhibit a high PL QY (>80%) and excellent stability in aqueous solution. For in vitro diagnostics, we used the InP QDs for AFP antigen detection based on the QDs‐FLISA procedure. A 5.8‐fold boost in the detection sensitivity (∼0.58 ng ml^−1^), and a wide detection range were achieved using the QDs‐2‐based antibody probe. Then, the InP QDs antibody probes were used for in vivo and in vitro tumor‐targeted imaging. The results showed that these probes were virtually non‐cytotoxic to cells cultured, and can target HepG2 cells and liver cancer tumors for recognition and labeling. These findings demonstrate the potential of InP QDs for in vivo tumor‐targeted imaging in cancer diagnosis and other clinical applications.

## 4 EXPERIMENTAL SECTION

Details of the reagents, instruments, and other preparation processes are provided in the Supporting Information.

## CONFLICT OF INTEREST

The authors declare no conflict of interest.

## ETHICS STATEMENT

All animal procedures were performed in accordance with the Guidelines for the Care and Use of Laboratory Animals of Henan University and experiments were approved by the Biomedical Research Ethics Sub‐Committee, Henan University (No. HUSOM2022‐094).

## Supporting information

Supporting Information

## Data Availability

All data related to this study are present in the article and in the Supporting Information. Any other data associated with this work are available from the corresponding authors upon request.
