# Supplementary material for: Aminophosphate precursors for the synthesis of near‐unity emitting InP quantum dots and their application in liver cancer diagnosis
Source: Exploration (Beijing). 2022 Jul 15;2(4):20220082. doi: 10.1002/EXP.20220082 (PMC10190939; doi:10.1002/EXP.20220082)
Supplement: Supplementary file 1 — Supporting Information [file EXP2-2-20220082-s001.doc]

Supporting Information

**Aminophosphate Precursors for the Synthesis of Near-Unity Emitting InP Quantum Dots and Their Application in Liver Cancer Diagnosis**

*Yanbin Zhang,^†^ Yanbing Lv,^†^ Lin-Song Li, Xue-Jie Zhao, Mei-Xia Zhao* and Huaibin Shen**

1. **Materials and Methods**

**1.1 Materials**

Indium chloride (InCl_3_, 99.99%, powder), zinc chloride (ZnCl_2_, 99.95%, powder), selenium (Se, 99.99%, powder), sulfur (S, 99.998%, powder), oleylamine (OAm, 98%), 1-octadecene (ODE, 90%), and tris(diethylamino)- phosphine ((DMA)_3_P, 97%) were purchased from Shanghai Aladdin Reagent Company. 3-mercaptopropionic acid (3-MPA), 3-(4,5-dimethyl-2-thiazol)-2,5-diphenyltetrazolium bromide (MTT), 1640 medium powder, DMEM medium powder, trypsin, and fetal bovine serum were analytical grade products purchased from Beijing Chemical Reagent Co. Calf serum, bovine serum albumin (BSA), and casein were purchased from Sigma-Aldrich. NaOH, HCl, KCl, NaCl, CaCl_2_, NaHCO_3_, Na_2_CO_3_, Na_2_HPO_4_, KH_2_PO_4_, H_3_BO_3_, Na_2_B_4_O_7_⋅10H_2_O, Tris, Hepes, and Tween-20 were acquired from Shanghai Sangon Ltd (Shanghai, China). Hexane (analytical grade), 1-butanol (analytical grade), n-octane (analytical grade), acetonitrile (analytical grade), and ethanol (analytical grade) were obtained from Beijing Chemical Reagent Ltd (Beijing, China). N-Hydroxysulfosuccinimide (sulfo-NHS), 1-ethyl-3-(3-(dimethylamino) propyl) carbodiimide (EDC), and the 96-well microplate were purchased from Thermo Fisher Scientific (USA). Mouse anti-AFP monoclonal coating antibody (AFP1 mAb, 4F16-4A3), mouse anti-AFP monoclonal labeling antibody (AFP2 mAb, 4F16-5H7), and AFP antigen were obtained from HyTest Ltd.

1.2 Synthesis of InP/ZnSe/ZnS core-shell QDs

Preparation of precursors:

Se-ODE precursor: A mixture of Se (0.31 g, 4 mmol) and ODE (10 mL) was loaded into a 10 mL single-mouth vial and sonicated.

SeS-ODE precursor: A mixture of Se (0.156 g, 2 mmol), S (0.064 g, 2 mmol), and ODE (10 mL) was loaded into a 10 mL single-mouth vial and sonicated.

Preparation of InP/ZnSe/ZnS QDs:

Firstly, 0.25 mmol of InCl_3_, 4 mmol of ZnCl_2_, and 8 mL of OAm were placed in a 50 mL three-necked flask, which was vented with argon at 120 °C for 1 h and then heated to a temperature of 180 °C. At this temperature, 0.25 mL of (DMA)_3_P was swiftly injected into the reaction flask, and it reacted for 30 min to realize InP nucleation. Following the synthesis of InP nuclear QDs, the reaction temperature was subsequently raised to 260 °C. Se-OAm (0.4 M) was added dropwise at a rate of 0.2 mL/5 min to grow the ZnSe layer. The total addition amount was 3 - 4 mL with photoluminescence (PL) wavelength > 630 nm. The reaction temperature was subsequently raised to 280 °C, and the ZnS layer was grown by adding S-OAm (0.4 M) dropwise at a rate of 0.2 mL/5 min. The total volume added was 3 - 4 mL with final PL observed at approximately 620 - 625 nm.

1.3 Ligand exchange of InP/ZnSe/ZnS QDs

The prepared InP/ZnSe/ZnSeS/ZnS QDs were extracted three times with hexane-ethanol and then dissolved in n-octane to a concentration of 10 mg mL^-1^. Then, we took 4 mL of the QDs dissolved in octane, added 4 mL/ammonia, adjusted the pH to 11, added 2 mL butanol and 1 mL 3-MPA, and stirred for 1 h at 60 °C (butanol was added to accelerate the transfer of QDs to the aqueous solution.^[1]^ After a completely layered solution was obtained, the aqueous solution of QDs in the lower layer was placed in a 50 mL centrifuge tube and purified by adding 30 mL of acetonitrile by centrifugation.

1.4 Photochemical processing

The purified InP QDs were re-dissolved in 5 mL water, 0.8 mmol ZnCl_2_, and 0.8 mL 3-MPA. The solution was then stirred at 75 °C for 1 h and irradiated by Ultraviolet (UV) light for 40 min. Finally, the QDs were purified by acetonitrile.

1.5 Characterization of InP QD

Ultraviolet-visible (UV-vis) spectroscopy and PL measurements were performed using an Ocean Optics spectrophotometer (model PC2000-ISA). TEM studies were performed using JEOL JEM-2100 and FEI Tecnai G2 F20 S-TWIN (equipped with an EDS detector from EDAX) electron microscopes both operating at 200 kV. All QYs data are absolute QYs and were collected by JY HORIBA FluoroLog-3 fluorescence spectrometer coupled with an integrating sphere. First, the samples were dissolved in hexane or water to obtain the clear and transparent solution. In order to ensure the dispersibility, the QDs solutions were treated with ultrasonication before the measurement. Second, the concentrations of sample solutions were determined by the UV-vis absorption. The optical density (OD) values of the sample solutions were set the same in the range of 0.02-0.05 at the first exciton absorption peak. Then, the sample solutions were measured by the JY HORIBA FluoroLog-3 fluorescence spectrometer and the absolute PL QYs were obtained from the corresponding software. The scattering absorption does not affect the accuracy of PL QY measurement, when the absorbance values of all sample solutions were kept below 0.05. Time resolved fluorescence spectra were obtained based on the time-correlated single-photon counting method using a JY HORIBA FluoroLog-3 fluorescence spectrometer with a 405 nm pulsed (ps) diode laser, whose repetition rate was 1 MHz and pulse duration was ~200 ps. The Fourier transform infrared (FTIR) spectra were obtained on an AVATAR360FT-IR spectrometer. The elements (In, Zn) in some of the samples were also determined by inductively coupled plasma (ICP) spectrometry (SPS-4000, SII). For thermogravimetric analysis (TGA), a sample with an approximate weight of 10 mg was placed in a thermal gravimetric analyzer and heated up to 900 °C at a rate of 5 °C/min to analyze the lost mass. The hydrodynamic diameters and zeta potentials of the QDs were obtained using a Zetasizer (Nano ZS, Malvern Instruments, U.K.). To collect the PL intensity of single QDs, the concentration of QDs solution should be low enough. First, the purified QDs solution was diluted by the poly(methyl methacrylate) (PMMA) solution (PMMA: toluene = 5wt%, magnetic stirring at 45 ℃ for 24 h). Then, the diluted QDs solution was spun and cast onto a fused silica substrate at 5,000 rpm for 20 s. The 405 nm output of a 5 MHz, picosecond diode laser or the 400 nm output of a CW laser was used as the excitation source. The laser beam was focused onto the sample substrate by an immersion-oil objective (NA = 1.4). The PL signal of a single QDs was collected by the same objective and sent through a 0.5 m spectrometer to a charge coupled-device camera for the PL spectral measurements. The PL signal of a single QDs can be alternatively sent through a nonpolarizing 50/50 beam splitter to 4 two avalanche photodiodes (APDs) in a time-correlated single photon counting (TCSPC) system with a time resolution of ∼250 ps. The TCSPC system was operated under the time-tagged, time resolved mode so that the arrival times of each photon relative to the laboratory time and the laser pulse time could both be obtained, which allowed us to plot the PL time trajectory.

1.6 Preparation of InP-antibody constructs probes

Firstly, 300 μL of aqueous InP QDs (10 mg mL^-1^) were dispersed in 750 μL sodium borate buffer (5 mM, pH 7.2, BS buffer) in a 1.5 mL-centrifuge tube. Subsequently, 50 μL of 0.226 M sulfo-NHS and 50 μL of 0.09 M EDC were added into the reaction solution and then activated to react for 10 min at 4 °C under ultrasonic vibration. Next, the supernatant was discarded to remove unreacted reagents by centrifugation at low temperature (4 °C). The mixture was re-dissolved in 400 μL BS buffer (5 mM, pH 8.0). Then, 20 μL AFP2 antibodies were added to the solution, and the resulting solution was incubated at 37 °C for 3 h. Afterwards, the mixture was blocked with 1 wt% casein solution, and the active sites were stopped with 12 μL ethanolamine. The QDs-antibody by centrifugation twice at low temperature (4 °C) for 30 min to remove the unlabelled antibodies. Finally, the InP QDs-antibody probes were stored in 50 μL BS solution (5 mM, pH 8.0) for the subsequent tests.

1.7 QDs-based fluorescence-linked immunosorbent assay (QDs-FLISA) process

For the detection of AFP antigens, the coating AFP1 antibodies (5.8 mg mL^-1^) were diluted by carbonate-bicarbonate buffer (0.05 M, pH 9.6, CB buffer) and incubated at 4 °C overnight. Then, the excess binding sites were blocked with BSA (0.5 wt%) in 0.01 M PBS (pH 7.4) incubating overnight at 4 °C after removing the excess coating antibody by washing three times with wash buffer (10 mM PBS containing 0.05% Tween-20, PBS-T). The microplate was dried in a constant temperature and humidity chamber for 24 h and then stored at 4 °C until further use. The standard solution was prepared by diluting AFP stock solution (1 μg mL^-1^) to final concentrations of 1, 2, 5, 10, 20, 50, 100, 200, 500, and 1,000 ng mL^-1^ with the sample buffer (10% calf serum (v/v) in 0.01 M PBS). Next, 100 μL/well of the standard antigens were incubated at 37 °C for 30 min, which was followed by five washes with the wash buffer (PBS-T). For subsequent operation, 100 μL of the QDs-antibody probes were added in the corresponding wells and incubated at 37 °C for 30 min. After five washes with a wash buffer (PBS-T), the PL intensity of each well in the plate was automatically read out by SpectraMax-i3 when the excitation wavelength was 450 nm.

The limit of detection (LOD) is one of the key parameters for immunoassay. Here, we tested the black wells (the negative control samples) for 20 times simultaneously. The LOD was calculated as follows: LOD = 3SD/Slope, where SD is the standard deviation (SD) of black well, and Slope is the slope of the low-concentration calibration plot.^[2]^

1.8 Cytotoxicity evaluation

SNU-739, Hep-G2, HL-7702, and A549 cells in a logarithmic growth phase were seeded in 96-well plates at a density of 8 × 10^3^ cells per well. After 24 h of culture in an incubator, the fluorescent probes at final concentrations of 20, 50, 100, 200, 500 μg mL^-1^ were added to each well. After 48 h of incubation, 50 μL MTT was added and incubated for 4 h. Next, 100 μL DMSO was added to dissolve the resultant formazan crystals. The absorbance at 570 nm of each well was measured, and the cell inhibition rate was calculated as follows:

Cell inhibition rate = (mean optical density (OD) value of negative group – mean OD value of experimental group) / (mean OD value of negative group - average OD value of blank group) × 100%.

1.9 Confocal laser scanning microscopy (CLSM) of InP-AFP-Ab probes uptake by cells

The Hep-G2 cells were seeded into laser confocal plates and cultured for 24 h. After co-incubation with a 500 μL aliquot of 10 μg mL^-1^ InP-AFP-Ab probes for 6 h, the uptake was observed by CLSM.

1.10 Ex-vivo imaging of the major organs of tumor-bearing mice

To assess the *in vivo* biodistribution of the InP-AFP-Ab probes, the probes were injected into mice through the tail vein, and 1, 2, 4, 6, 8, and 12 h after injection, the mice were dissected to obtain hearts, livers, spleens, lungs, kidneys, and tumours using a small animal live imaging system (IVIS Lumina XRMS Series III) for fluorescence imaging.

1. **Results and discussion**


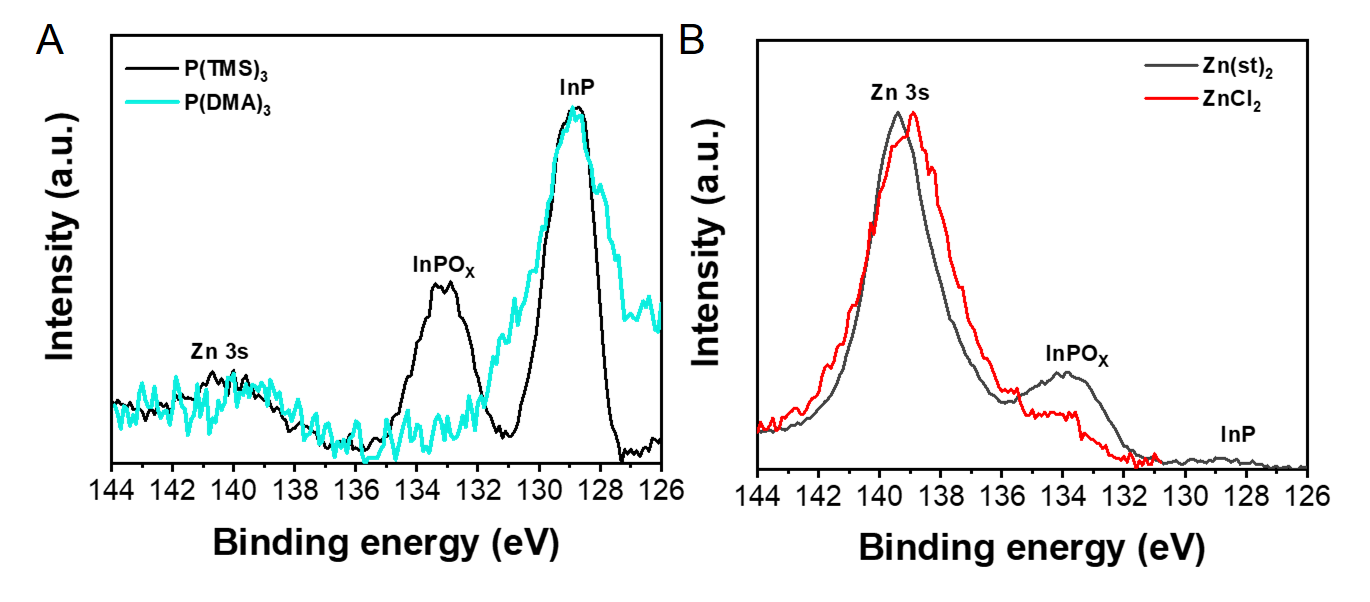


**Figure S1.** (A) XPS spectra of P 2p of InP cores prior to ZnSe growth (black: (TMS)_3_P, cyan: (DMA)_3_P); (B) XPS spectra of P 2p after growth ZnSe layers using different zinc precursors (black: Zn(st)_2_, red: ZnCl_2_).


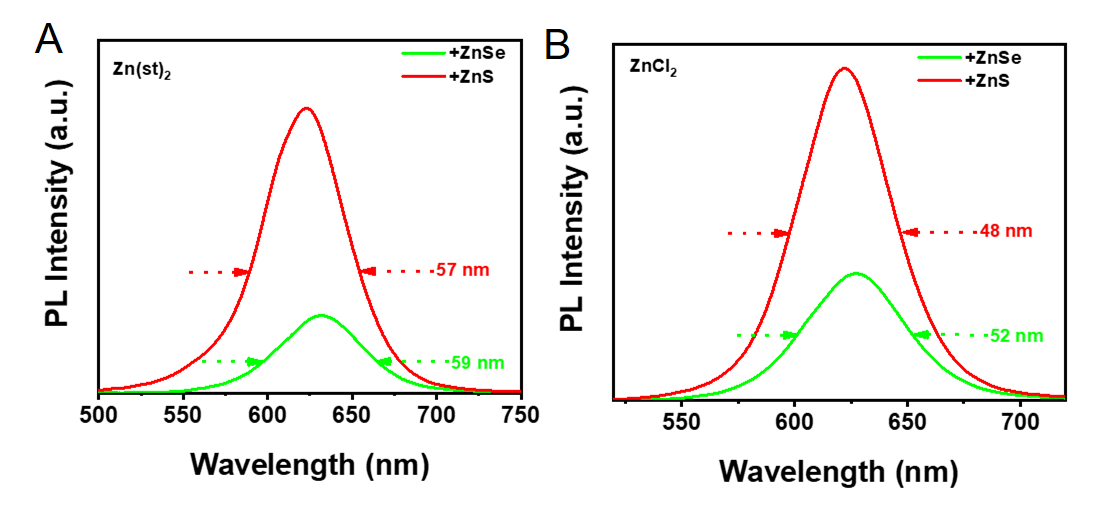
 **Figure S2.** The PL spectra after the same shells was grown with (A) Zn(st)_2_ and (B) ZnCl_2_ as zinc sources, respectively, and the PL intensity was not normalized.


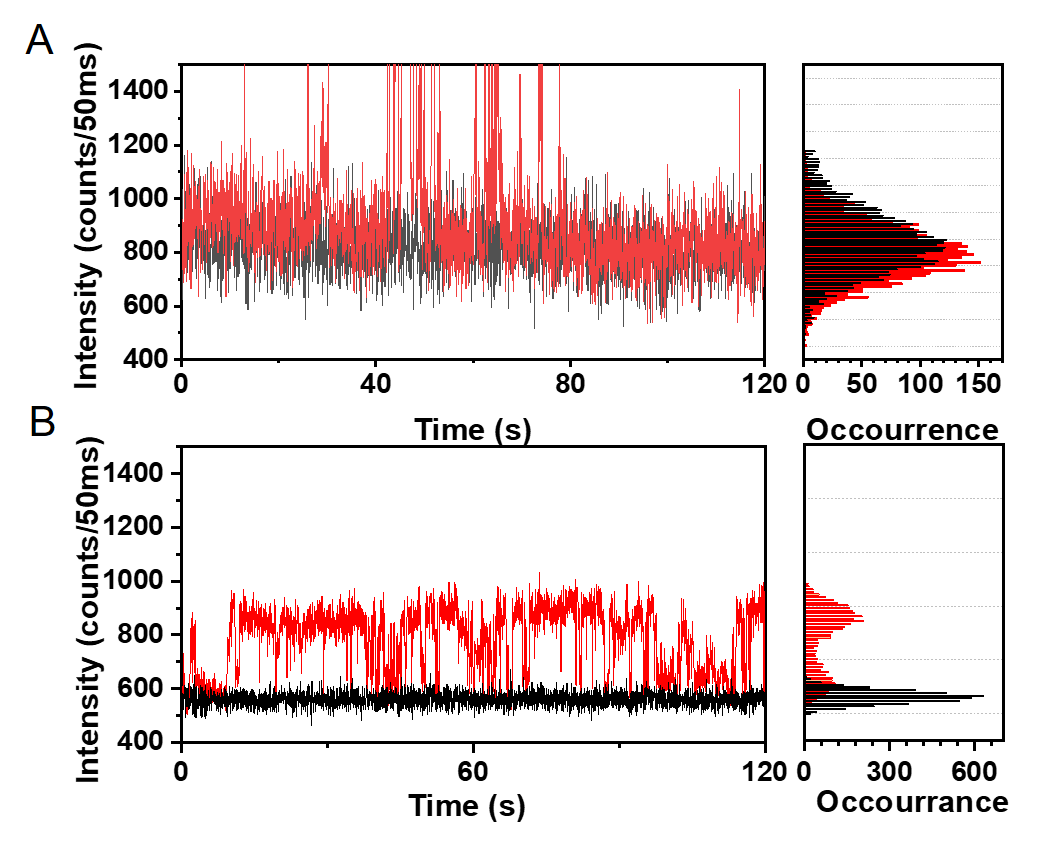


**Figure S3.** PL intensity trajectory of single (A) InP QDs and (B) InP/ZnSe core/shell QDs. The red and black traces are the PL intensities of a single QD and the corrected background noise intensities, respectively.


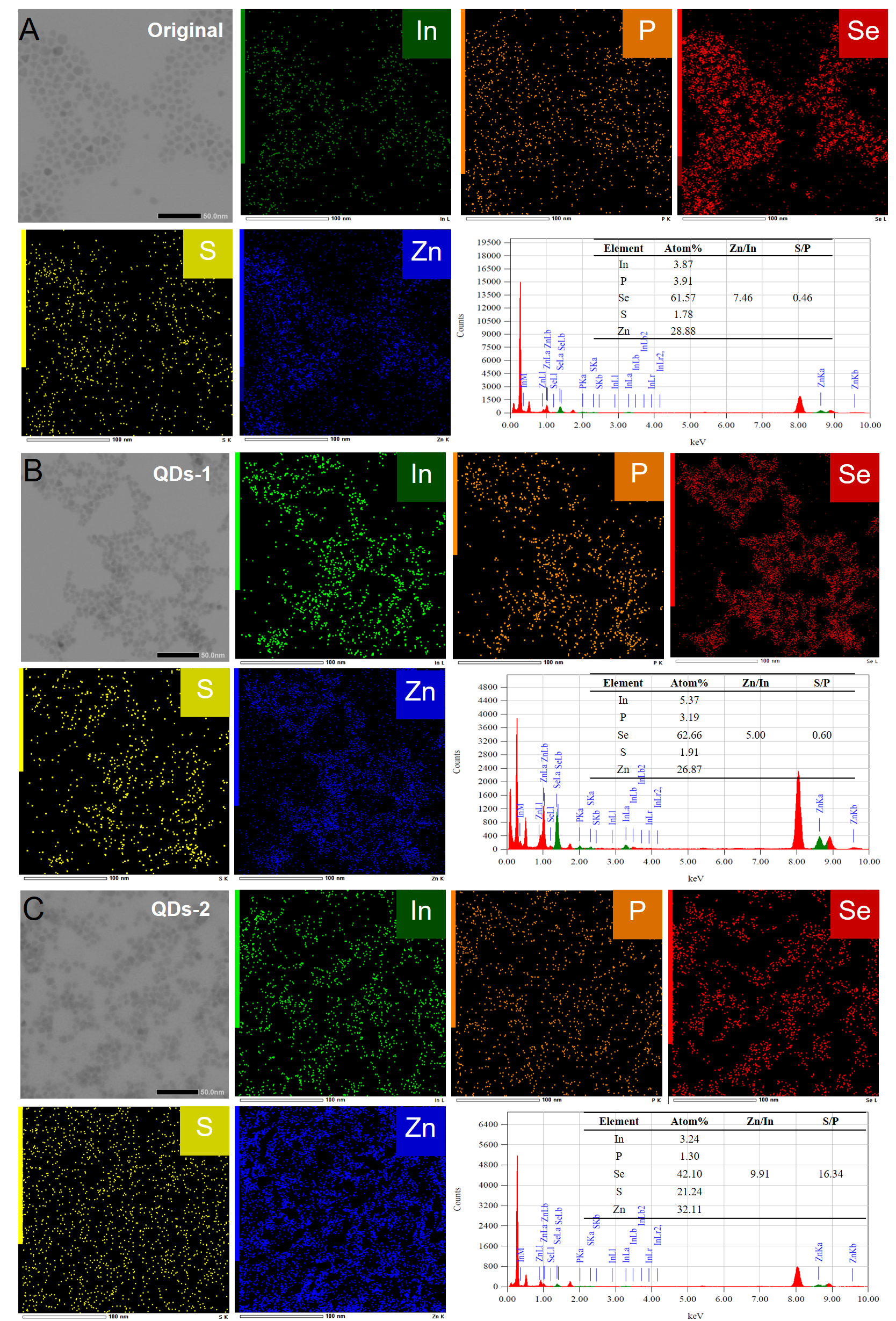


**Figure S4**. Elemental dispersive X-ray spectroscopy (EDS) elemental maps showing the distribution and content of each element in the QDs: (A) Original, (B) QDs-1, and (C) QDs-2.


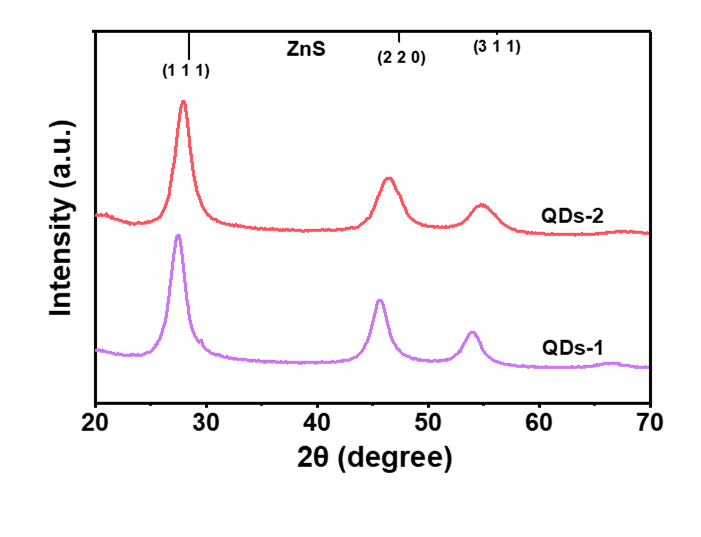


**Figure S5.** X-ray diffraction (XRD) patterns of QDs-1 and QDs-2. Standard XRD stick patterns of bulk zincblende ZnS is also provided.


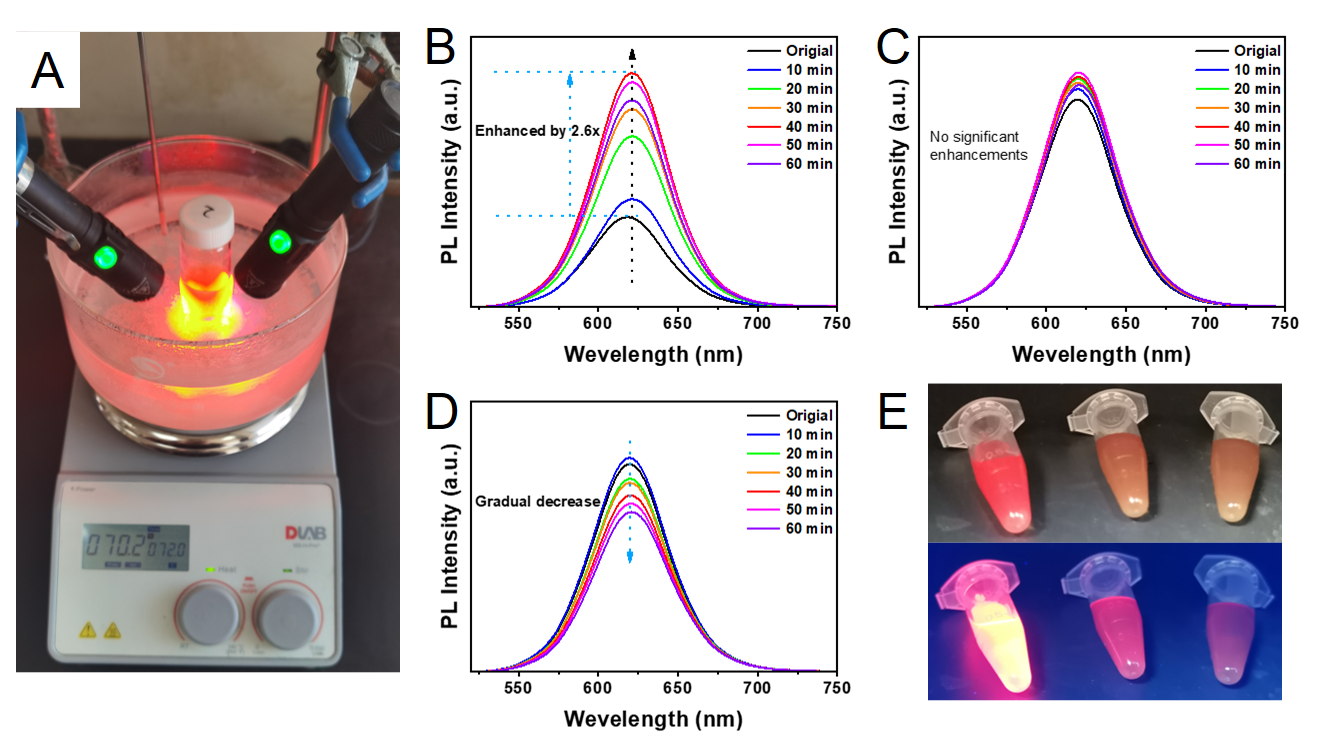


**Figure S6.** (A) Diagram of the reaction apparatus, where the temperature is 55-60 °C during ligand exchange and 70-75 °C during photochemical processing. (B) Effect of UV irradiation time on the PL intensity during photochemical processing; (C) no UV irradiation; (D) replacement of CaCl_2_ with ZnCl_2_, (E) photographs under natural light and UV light corresponding to the three conditions. The figures from left to right correspond to the experimental conditions in Figures b, c, and d, respectively.

**
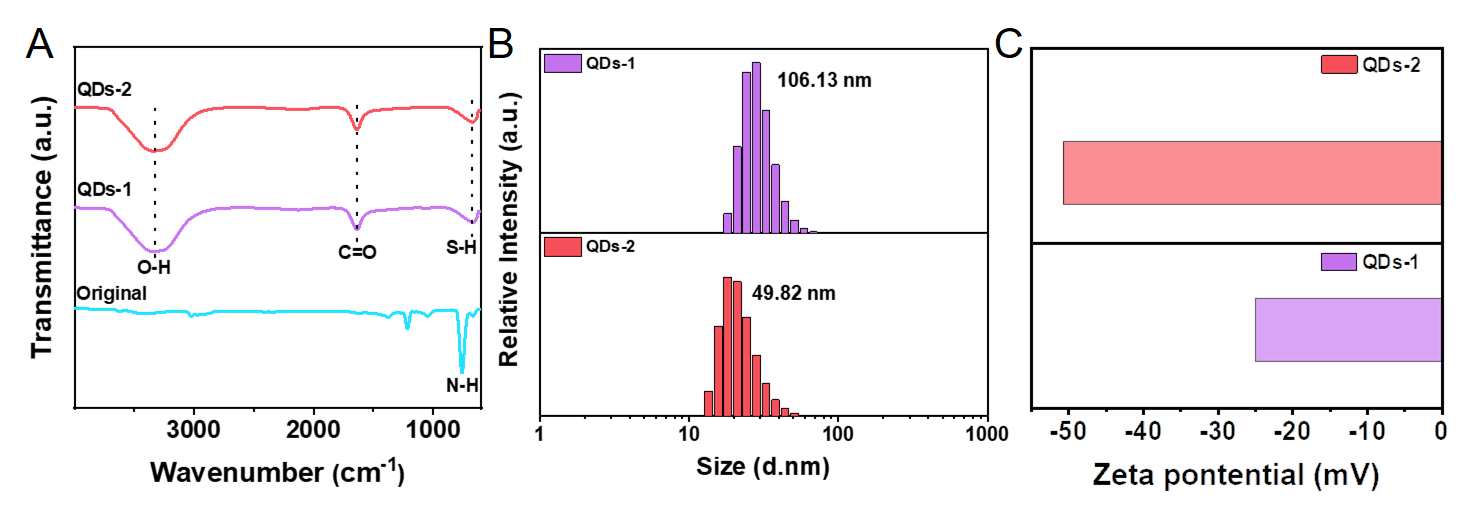
**

**Figure S7.** (A) FTIR spectra, (B) zeta potentials, and (C) dynamic light scattering analysis of QDs-1 and QDs-2.


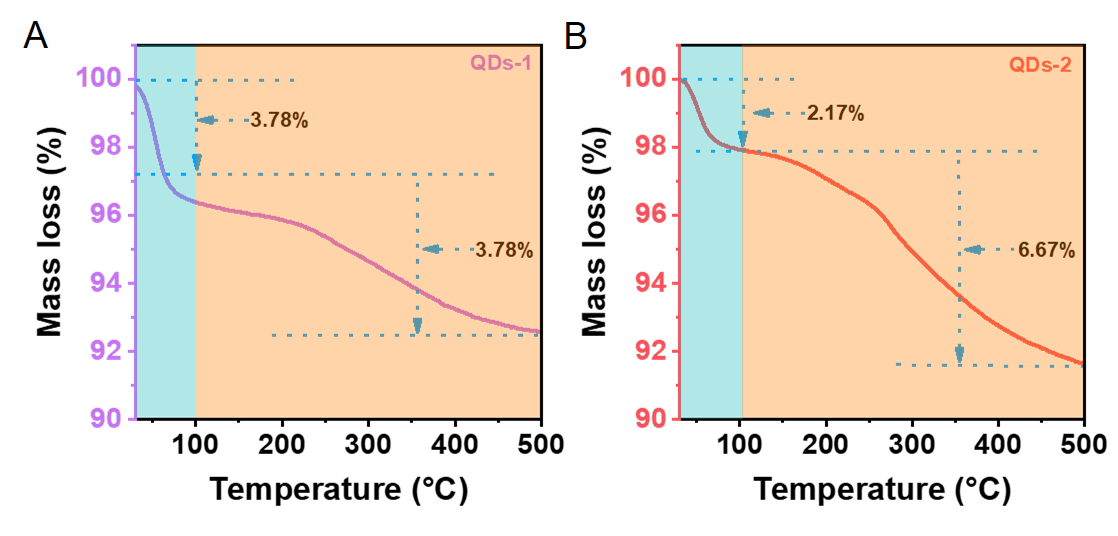


**Figure S8.** (A) and (B) mass percent versus temperature thermograms for TGA of QDs-1 and QDs-2.

Here, we use thermal gravimetric analysis (TGA) to quantify the ligand density on the surface of QDs. As shown in Fig. S6A and B, when the temperature is risen to 100 °C, the mass fractions lost by QDs-1 and QDs-2 are 3.78% and 2.17%, respectively. Considering the possible residual moisture in the sample, we have not calculated the weight lost between 0 - 100 °C and only considered the region between 100 - 500 °C. The calculation results suggest that the mass fractions lost by QDs-1 and QDs-2 are 3.78% and 6.67%, respectively. Based on the calculation of Lemuel Tong *et al.* and using Equation (1), the surface ligand densities of QDs-1 and QDs-2 are calculated to be 1.57 and 5.36 ligands per nm^-2^, respectively.^[3]^


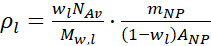
 (1)

where *w_l_* is the mass fraction of the ligand, *N_Av_* is the Avogadro’s constant, *M_w,l_* is the molecular weight of the ligand, (1-*w_l_*) is the mass fraction of the QDs, and *A_NP_* is the surface area of the QDs. The density of QDs (*ρ_NP_*) is calculated by the elemental proportion based on EDS, and the corresponding mass of individual QDs (*m_NP_*) can be calculated as follows:


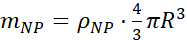
 (2)

where *R* is the radius of the QDs.


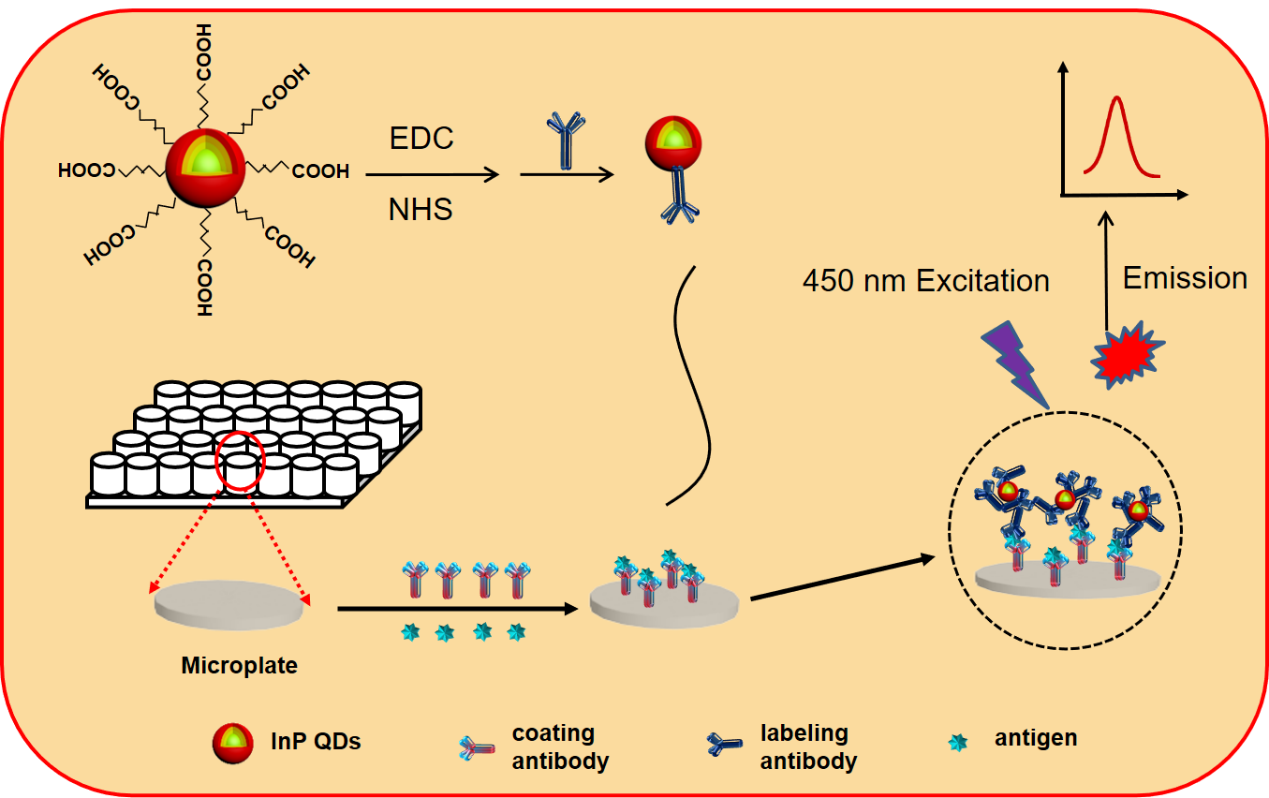


**Figure S9.** Schematic diagram of the detection of AFP antigen based on QDs-FLISA.


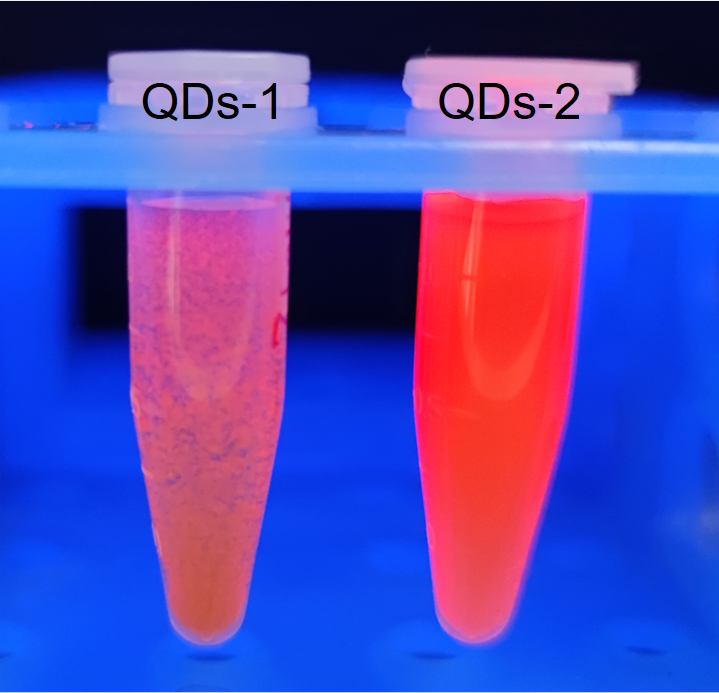


**Figure S10.** Pictures under UV irradiation after activation by adding EDC and NHS. It is clear that QDs-1 exhibit significant agglomeration.


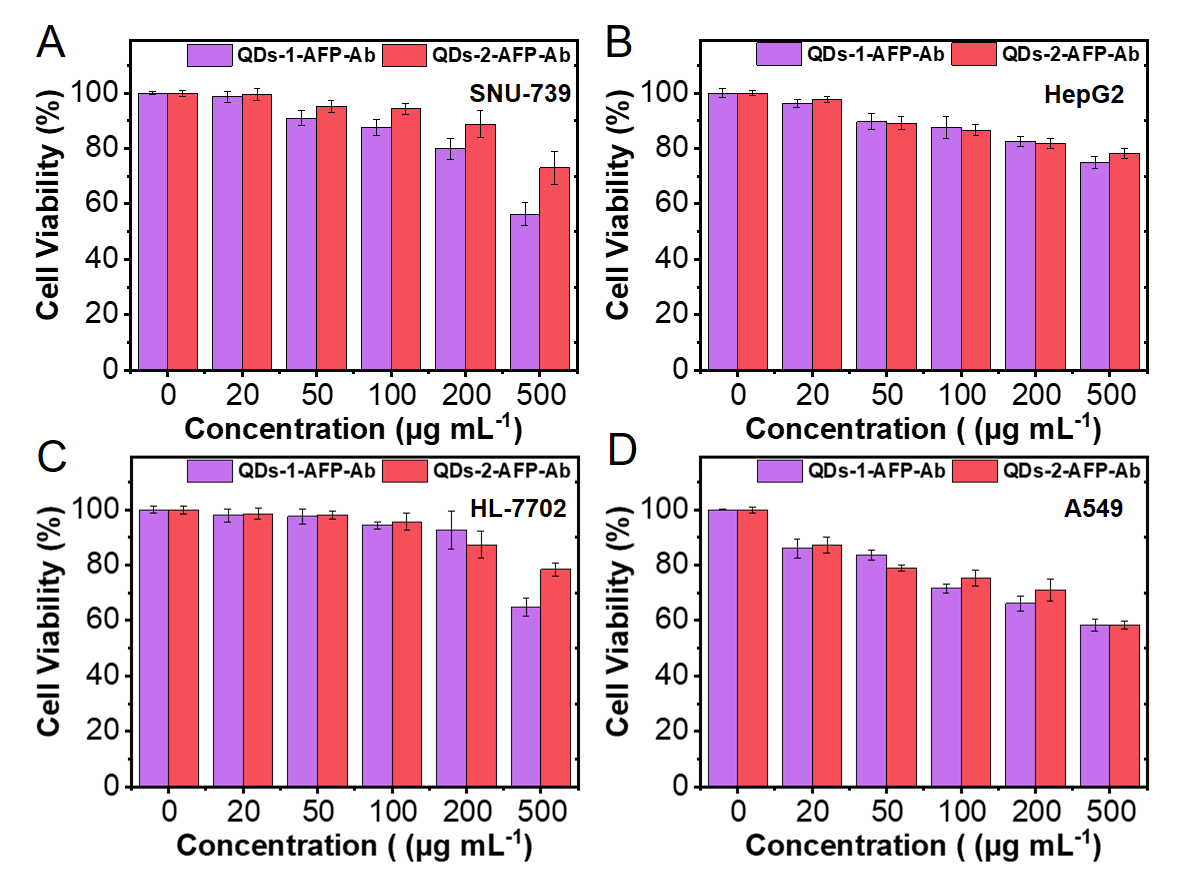


**Figure S11.** *In vitro* cell viability of cells treated with varying concentrations of QDs probe for 24 h. The percentage cell viability of the treated cells is calculated relative to that of the untreated cells (with arbitrarily assigned 100% viability).


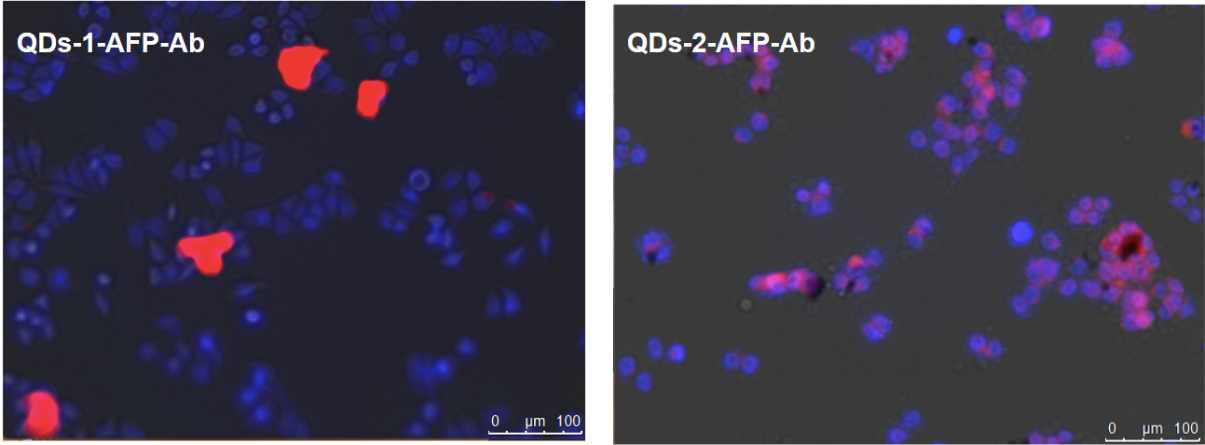


**Figure S12.** Fluorescent Inverted Micrograph of the probe after 6 h incubation with HepG2 cells.


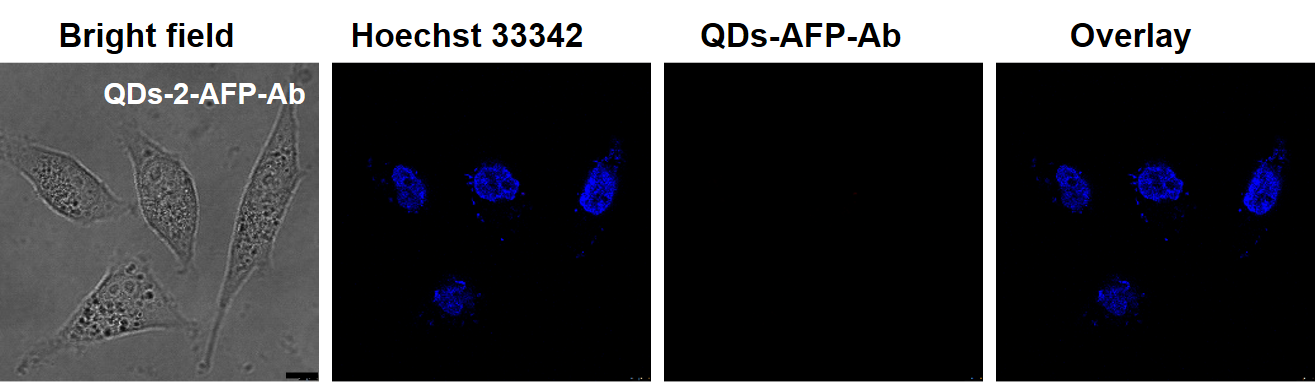
 **Figure S13.** The control studies by using MCF-7 cells, an AFP-negative human breast cancer cell line, no significant QDs fluorescence was observed. Scale bar: 8 μm.

**Table S1.** Elemental analysis (ICP) and calculation of Zn/In ratio.

| Sample | Element | Elemental content (μg L^-1^) | Zn/In |
| --- | --- | --- | --- |
| QDs-1 | Zn | 140338.0 | 4.02 |
|  | In | 34876.4 |  |
| QDs-2 | Zn | 266048.0 | 7.13 |
|  | In | 37317.9 |  |

**Table S2.** Comparison of different reported methods for AFP detection.

| Material | Method | Range (ng mL^-1^) | LOD (ng mL^-1^) | References |
| --- | --- | --- | --- | --- |
| CdTe QDs & gold nanoparticles | Förster resonance energy transfer | 0.5-45 | 0.4 | [4] |
| CdSe/ZnS QDs | Immunochromatographic test strip | ----- | 3 | [5] |
| CdSe QDs | Immunochromatographic test strip | ----- | 1 | [6] |
| Magnetic Nanoparticles | Electrochemiluminescent Immunoassay | 1 - 200 | 0.32 | [7] |
| InP QDs | QDs-FLISA | 1 - 1,000 | 0.58 | This work |

References

[S1] C. Li, M. Ando, H. Enomoto, N. Murase, *J. Phys. Chem. C* 2008, **112**, 20190.

[S2] Y. Lv, Y. Yuan, N. Hu, N. Jin, D. Xu, R. Wu, H. Shen, O. Chen, L.S. Li, *ACS Appl. Nano Mater.* 2021, **4**, 2855.

[S3] L. Tong, E. Lu, J. Pichaandi, P. P. Cao, M. Nitz, M. A. Winnik, *Chem. Mater.* 2015, **27**, 489.

[S4] L. Zhou, F. Ji, T. Zhang, F. Wang, Y. Li, Z. Yu, X. Jin, B. Ruana, *Talanta* 2019 **197**, 444.

[S5] C. Wang, F. Hou, Y. Ma, *Biosens. Bioelectron.* 2015, **68**, 156.

[S6] Q. Yang, X. Gong, T. Song, J. Yang, S. Zhu, Y. Li, Y. Cui, Y. Li, B. Zhang, J. Chang, *Biosens. Bioelectron* 2011, **30**, 145.

[S7] Z. J. Huang, W. D. Han, Y. H. Wu, X. G. Hu, Y. N. Yuan, W. Chen, H. P. Peng, A. L. Liu, X. H. Lin, *J. Electroanal. Chem.* 2017, **785**, 8.
